# Combined morphological and multi-omics analyses to reveal the developmental mechanism of *Zanthoxylum bungeanum* prickles

**DOI:** 10.3389/fpls.2022.950084

**Published:** 2022-08-22

**Authors:** Kexing Su, Jiaqian Sun, Jun Han, Tao Zheng, Bingyin Sun, Shuming Liu

**Affiliations:** ^1^College of Science, Northwest Agriculture and Forestry University, Xianyang, China; ^2^Powerchina Northwest Engineering Corporation Limited, Xi’an, China; ^3^Shaanxi Union Research Center of University and Enterprise for River and Lake Ecosystems Protection and Restoration, Xi’an, China; ^4^Forestry and Grassland Bureau of Xunhua County, Qinghai, China; ^5^Department of Ecological Engineering, Yangling Vocational and Technical College, Xianyang, China

**Keywords:** *Zanthoxylum bungeanum*, prickles, resembling abscission zone, vacuolar deposition, transcriptome, metabolome

## Abstract

*Zanthoxylum bungeanum* Maxim. as an important economic forest, its epidermis bears prickles which complicate the harvesting process and increase the labor costs. To explore the developmental mechanism of prickles, three varieties of *Zanthoxylum bungeanum* (PZB, SZB, GSZB) were selected for morphological and multi-omics analyses. The absorption spectra of prickles and stems were detected using Fourier-transform infrared spectroscopy (FTIR), and they were found different at 1617, 1110, 3319, and 1999 cm^–1^. The morphology of prickles and stems were observed using light microscopy and transmission electron microscopy (TEM). The growth direction of cells on the prickle side and stem side were perpendicular to each other, and there was a resembling abscission zone (RAZ) between them. The vacuolar deposits of prickle cells were much more than stem cells, indicating that the lignification degree of prickles was higher than stems. In addition, 9 candidate genes (*ZbYABBY2*, *ZbYABBY1*, *ZbYABBY5*, *ZbWRKY*, *ZbLOG5*, *ZbAZG2*, *ZbGh16*, *ZbIAA33*, and *ZbGh16X1*) were screened out and validated base on transcriptome and qRT-PCA. As well as, 30 key metabolites were found related to prickle development base on metabolome analysis. Among them, 4-hydroxy-2-oxopentanoate, trans-2-hydroxy-cinnamate, trans-cinnamate, polyhydroxy-fatty acid, 10,16-dihydroxypalmitate, cinnamic acid were related to the biosynthesis of cutin, suberine and wax. Indole-3-acetate, tryptamine, anthranilate, fromylanthranilate, N6-(delta2-isopentenyl)-adenine were related to plant hormone signal transduction. Generally, this is the first study to reveal the developmental mechanism of prickles. The results of this study lay the foundation for the breeding of non-prickle *Zanthoxylum bungeanum*.

## Introduction

Plants with spines, thorns or prickles are widely spread in nature (Simpson, 2019; Airoldi and Glover, 2020). These sharp pointed structures protect them from herbivores (Grubb, 1992; Simcha, 2016). In botanical terms, spines refer to modified leaves, thorns refer to modified stems, while prickles refer to modified epidermal cells (Steeves and Sussex, 1989; Huchelmann et al., 2017). In this study, our research object is prickles. The representatives of prickle-bearing plants including *Girardinia suborbiculata*, *Chorisia speciosa*, *Zanthoxylum bungeanum*, *Rubus corchorifolius*, and *Rosa chinensis* which suggests that prickles have evolved many times and represent an example of convergent evolution (Sussex and Kerk, 2001; Zhang et al., 2020).

**FIGURE 1:**
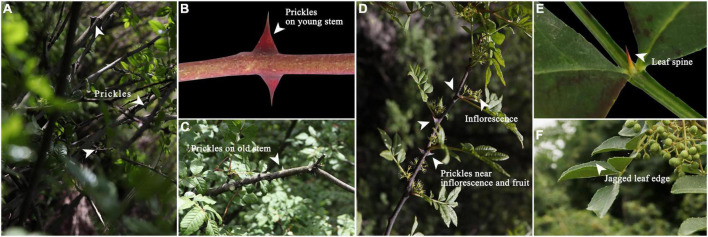
**(A)** Prickles; **(B)** Prickles on young stem; **(C)** Prickles on old stem; **(D)** Prickles near inflorescence; **(E)** Leaf spine; **(F)** Jagged leaf edge.

*Zanthoxylum bungeanum* (Rutaceae), a drought-tolerant shrub, has been widely cultivated for its medicinal and edible value (Yang et al., 2013; Ma et al., 2019; Liu et al., 2020). Its pericarps, branches, roots and leaves are all medicine (Sun et al., 2019, 2020), which has been used to treat asthma (Tang et al., 2014), obesity (Wang et al., 2019, 2020), colitis (Zhang et al., 2017), diabetes (Zhang et al., 2021), and eczema (Su et al., 2020). As the native of *Zanthoxylum bungeanum*, Chinese cultivation area and output are both the largest in the world (Li et al., 2015; Chen et al., 2019; Sheng et al., 2020). However, evolution and natural selection have led the epidermis of *Zanthoxylum bungeanum* to bear prickles (Coverdale, 2020), just as Figure 1 shows. These prickles make cultivation management difficult and at the same time result in high harvest costs (Appelhans et al., 2018; Fei et al., 2020). The same problem also plagues other prickle-bearing cash crops such as *Rosa chinensis*, *Vitis vinifera*, and *Rosa roxburghii* (Rajapakse and Arumuganathan, 2001; Debener and Linde, 2009; Wang et al., 2021).

Early research suggests that prickles are modified glandular trichomes that differentiate into prickle morphologies upon lignification (Kellogg et al., 2011). Until recently, only a few studies had been published about the mechanism of prickle development, but great progress had been made in trichome formation. In *Arabidopsis thaliana*, more than twenty genes are required to control the development of trichomes (Szymanski et al., 1998). The initial selection of trichome precursors requires the activity of both *GL1* and *TTG* genes (Herman and Marks, 1989; Payne et al., 1999). The *GL2* gene is required for subsequent phases of trichome morphogenesis (Chopra et al., 2019). And gibberellins could promote trichome formation by up-regulating *GL1* (Chien and Sussex, 1996; Perazza et al., 1998; Matias-Hernandez et al., 2016). In addition, *CPC*, *TRY*, *TCL1*, *ETC1, ETC2*, and *ETC3* are negative regulators of trichome initiation (Wada et al., 1997; Glover et al., 1998; Kirik et al., 2005; Wang et al., 2007). “Activator-inhibitor model” and “activator-substrate model” can be used to explain the mechanism of trichome development (Balkunde et al., 2010).

Except *Arabidopsis thaliana*, research on prickles of *Rosa roxburghii*, *Vitis vinifera*, and *Rosa chinensis* has also made some progress. Huang and his team proved that RrGL1 of *Rosa roxburghii* is an R2R3 MYB homolog which regulates trichome formation by interacting with GL3/EGL3 protein (Huang et al., 2019; Yan et al., 2021). In *Vitis vinifera*, Yin and Barba’s research validated and mapped a major QTL for trichome density (Barba et al., 2019; Yin et al., 2021). In *Rosa chinensis*, *RcGL1*, *RcMYB82*, *RcMYB61*, *RcCPC*, *RcTRY*, *RcGL3*, *RcTT8*, *RcMYC1*, *RcTTG1*, *RcTTG2*, *RcZFP5*, *RcGIS3*, *RcGIS2*, and *RcZFP1* genes were considered candidate genes to control prickle development (Bourke et al., 2018; Zhou et al., 2020). Although some progress has been made in the study of prickles, there is no research on *Z. bungeanum* prickles yet.

To explore the developmental mechanism of *Z. bungeanum* prickles, this study selected three varieties of *Z. bungeanum* as experimental materials. Using Fourier-transform infrared spectroscopy, optical microscopy, transmission electron microscopy, transcriptomics and metabolomics methods to explore the developmental mechanism of prickles from the perspective of morphological and multi-omics analysis. Our research not only revealed the submicroscopic structure of prickles, but also screened out candidate genes and metabolites, which provided a theoretical basis for the breeding of non-prickle *Zanthoxylum bungeanum*.

## Materials and methods

### Plant materials

**FIGURE 2:**
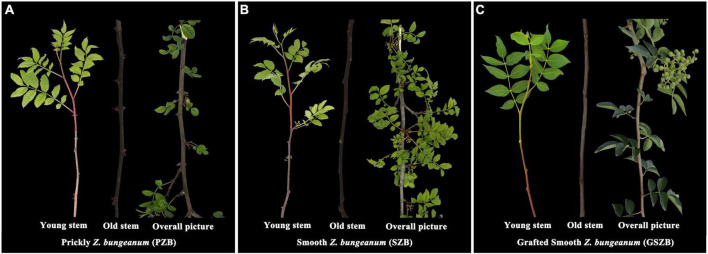
**(A)** Wild Prickly *Zanthoxylum bungeanum*; **(B)** Wild Smooth *Zanthoxylum bungeanum*; **(C)** Grafted Smooth *Zanthoxylum bungeanum*.

Three varieties of *Z. bungeanum* used for experiment were: Wild Prickly *Zanthoxylum bungeanum* (PZB), Wild Smooth *Zanthoxylum bungeanum* (SZB), and Grafted Smooth *Zanthoxylum bungeanum* (GSZB). Previous studies have shown that grafting can inhibit the expression of *Z. bungeanum* prickles, and the inhibition effect of grafting with young stems is more obvious than that with old stems. Therefore, we collected both young and old stems as experiment materials (three biological replicates per tissue), as shown in Figure 2.

The “Grafted Smooth *Zanthoxylum bungeanum*” was planted in Didian village (109.227158 E; 34.985359 N), The “Wild Prickly *Zanthoxylum bungeanum*” and “Wild smooth *Zanthoxylum bungeanum*” were picked in the Qinling Mountains (108.329167 E; 33.801945 N). These plant tissues were immediately frozen in liquid nitrogen and stored at −80°C for subsequent analysis.

### Fourier transform infrared spectroscopy

The plant tissues were divided into 14 groups (Supplementary Table 1). They were placed in an oven to dry (60°C, 24 h) and then ground to powder. The powder was mixed with KBr at a ratio of 1:100 and pressed into flakes. The Fourier-transform infrared spectroscopy (vertex70, Germany) was used to determine the absorption spectra of plant tissues, each sample repeated three times. Finally, data analysis was performed using OPUS software (BRUKER, Munich, Germany) (Hu et al., 2021; Teimouri et al., 2021).

### Preparation and observation of paraffin sections

The plant tissues were cut into small pieces (5mm × 5mm × 2mm) and fixed with FAA solution (38% Acetaldehyde 5 ml, 70% ethanol 90 ml, glacial acetic acid 5 ml, glycerol 5 ml) for 24 h. Then, they were subjected to the following dehydration procedure: in 70, 80, 90, 95, and 100% ethanol (each of 30 min), 1:1 mixture of xylene and absolute ethanol (30 min), xylene solution (twice, 20 min). Subsequently, they were embedded in paraffin wax and placed in a 38°C oven for 48 h. Then, they were cut into 8–10 μm sections using Leica microtome. The sections were stained with 0.05% toluidine blue (pH 4.3) and examined by Leica light microscope (Ma et al., 2021).

### Observation of transmission electron microscope

The young stem segments were fixed with 4% glutaraldehyde at 4°C for 12 h and washed four times with 0.1 M phosphate buffer (pH 6.8). Afterward, they were post-fixed with 1% osmium tetroxide and subjected to the following dehydration procedure: in 30, 50, 70, 80, 90, and 100% alcohol (twice, each of 10 min), and then embedded in epoxy resin. Later, the material was cut into 80 nm ultrathin sections using ultrathin microtome (EMUC7). The ultrathin sections were transferred to copper grids and post-stained with uranyl acetate and lead citrate. Observations were made with a TECNAI G2 SPIRIT BIO TEM at 120 kV (Naidoo et al., 2012; Munien et al., 2015).

### cDNA library construction, sequencing, and data analysis

The young stem bark of PZB, SZB, and GSZB was sent to Sangon Biotech (Shanghai, China) for library construction, quality control, and paired-end sequencing with Illumina HiSeq™ (Bolger et al., 2014). After sequencing, the clean reads were selected by removing low-quality sequences, and the transcripts were obtained by assembling clean reads using Trinity software (Wang et al., 2012; Okonechnikov et al., 2016). Then the transcripts were de-redundant to get unigenes. And unigenes were compared to public databases (NR, NT, KOG, Pfam, Swissprot, CDD, TrEMBL, GO, KEGG) using BLAST with an *e*-value threshold of 10^–5^.

### Identification and analysis of differentially expressed genes

To verify the transcription expression levels of all samples, transcripts per million (TPM) were used to quantify the expression level of genes. Differentially expressed genes (DEGs) between SZB, PZB and GSZB samples were identified using DESeq2, with | log_2_ fold change| > 2 and *q*-Value < 0.05. GO enrichment analyses were performed using clusterProfiler (Kanehisa and Goto, 2000; Tatusov et al., 2000; Marchler-Bauer et al., 2013; UniProt, 2015; Finn et al., 2016). Sequence alignment and phylogenetic analysis were performed on the homologous proteins sequences of candidate genes using MEGA software. And CD-Search was used to analyze conserved domains of homologous proteins.^1^

### Quantitative real-time polymerase chain reaction verification

The specific quantitative primers (Supplementary Table 2) were designed using Primer 5.0 software, and the candidate gene sequences were shown in Supplementary Table 3. The total ribonucleic acid (RNA) was extracted from young stem bark of PZB, SZB, and GSZB using TRIzol reagent (Invitrogen, Carlsbad, CA, United States). First-strand cDNA was synthesized using the PrimeScript™ RT Reagent Kit (TAKARA, Beijing, China) according to the manufacturer’s instructions. TB Green^®^ Premix Ex Taq™ (TAKARA, China) was used to perform qRT-PCR on an ABI StepOne Plus (Applied Biosystems, Foster, CA, United States) (Qin et al., 2020). Relative quantification of specific mRNA levels was performed using the cycle threshold 2^(–ΔΔCt)^ method with the *Zanthoxylum bungeanum* 18S rRNA gene (HG002512.1) as an internal control.

### Metabolomic profiling

The young stem bark of PZB, SZB, and GSZB were selected for metabolomic analysis. Extraction and analysis of differentially accumulated metabolites (DAMs) were performed by the Metware Biotechnology Co., Ltd. (Wuhan, China). Biological samples are freeze-dried by vacuum freeze-dryer (Scientz-100F) (Chen et al., 2013). Then the sample extracts were analyzed using an UPLC-ESI-MS/MS system (UPLC, SHIMADZU Nexera X2; MS, Applied Biosystems 4500 Q TRAP) (Chen et al., 2009; Fraga et al., 2010). DAMs were identified based on the thresholds | log_2_(fold change)| ≥ 1 and VIP (variable importance in project) ≥ 1 (Fu et al., 2021).

### Integrative analysis of transcriptome and metabolome

Genes and metabolites with a Pearson correlation coefficient (PCC) > | 0.8| and *P*-value < 0.05, were used to draw the nine-quadrant diagram and cluster heatmap (Jozefczuk et al., 2010).

## Results

### Differences in functional groups of prickle, bark, and stalk

**FIGURE 3:**
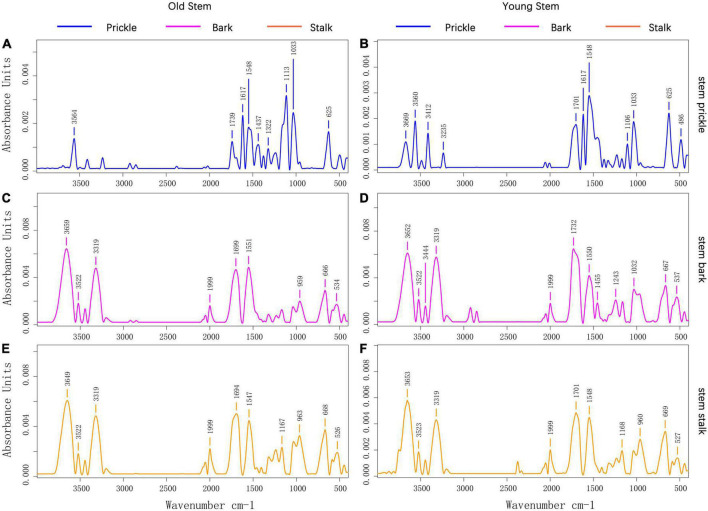
**(A)** Prickle’s absorption spectrum on old stems of PZB; **(B)** Prickle’s absorption spectrum on young stems of PZB; **(C)** Bark’s absorption spectrum on old stems of PZB; **(D)** Bark’s absorption spectrum on young stems of PZB; **(E)** Stalk’s absorption spectrum on old stems of PZB; **(F)** Stalk’s absorption spectrum on young stems of PZB.

The FTIR absorption spectrum of prickle was different from that of bark and stalk (Figure 3). The absorption spectrum of prickle had band at 1617 and 1110 cm^–1^, while the bark and stalk had strong band at 3319 and 1999 cm^–1^. According to the band correspondence, the associated -NH_2_ or -NH bond could cause a band to appear at 3319 cm^–1^, which means there were compounds contain such chemical bonds in bark and stalk. Besides the 1110 cm^–1^ band was caused by C-O-C stretching and symmetric vibration of the ester linkage, or -CH stretching in aromatic ring (Breub et al., 2010; Lupoi et al., 2015). In addition, the well-defined narrow band at 1617 cm^–1^ was attributed to C = C aromatic ring vibration, which indicated that the prickles contain a lot of aromatic compounds (Reyes-Rivera and Terrazas, 2017).

The FTIR absorption spectra of young and old stalk of PZB, SZB, and GSZB are shown in Supplementary Figure 1A, while the FTIR absorption spectra of young and old bark of PZB, SZB, and GSZB are shown in Supplementary Figure 1B. The results suggested that there was no significant difference in their functional groups between young and old stems, as well as between bark and stem. Therefore, we focused our research on prickle and young stems, which were significantly different, and explored them further later.

### Microstructure of prickles and young stems

**FIGURE 4:**
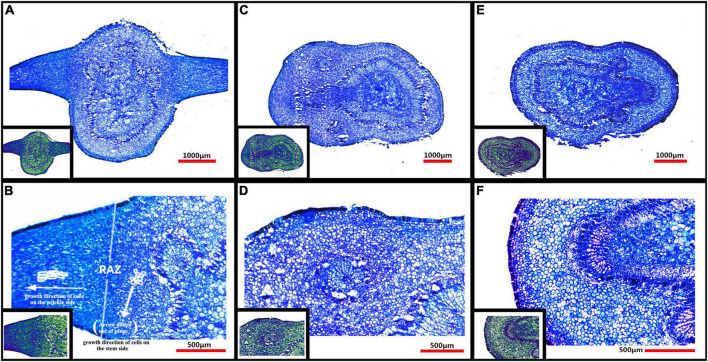
**(A)** Microstructure of PZB’s young stem and prickles; **(B)** Partial enlarged view of panel **(A)**; **(C)** Microstructure of SZB’s young stem; **(D)** Partial enlarged view of panel **(C)**; **(E)** Microstructure of GSZB’s young stem; **(F)** Partial enlarged view of panel **(E)**. The lower left corner of each image is its corresponding pseudo-color image.

From Figure 4A, it can be seen that there was no vascular bundle in prickles, which means that the prickles of *Zanthoxylum bungeanum* were different from thorns, and belonging to the category of prickles (Airoldi and Glover, 2020). Besides, the growth direction of cells on prickle side and stem side were perpendicular to each other (Figure 4B), and there was an obvious dividing line between them, called resembling abscission zone (RAZ). The finding of RAZ provided a good explanation for why prickles were easily peeled off from stems.

The microstructure of prickles and young stems of PZB, SZB, and GSZB were shown in Figure 4. It can be found that the parts with high degree of lignification were deeply stained, while the parts with low degree of lignification were lightly stained. This difference was more obvious in pseudo-color images, that the spicule cells were similar in color to xylem cells and epidermal cells, while the stem cells were similar in color to parenchyma cells. This result indicated that prickle cells were lignified earlier than stem cells.

### Ultra-microstructure of prickles and young stems

**FIGURE 5:**
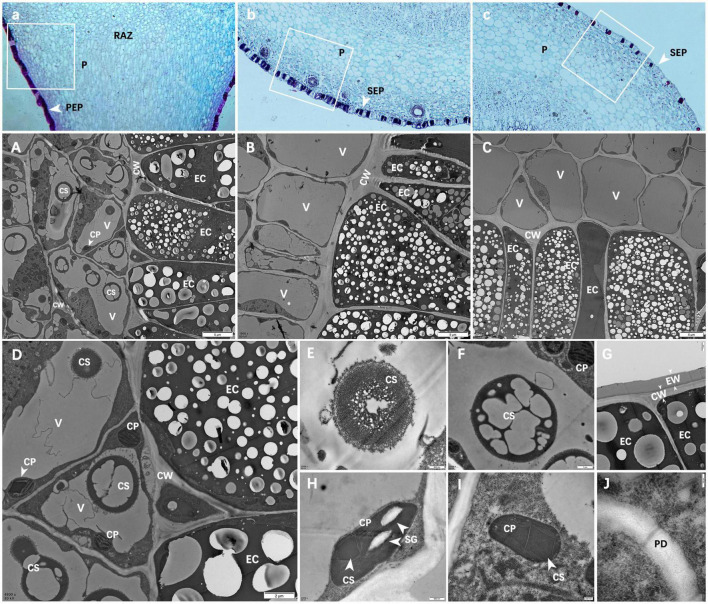
Ultra-microstructure of prickle and stem cells. **(A–C)** Are partial enlargements of the white boxes in panels **a–c**; **(A)** Epidermal cells of PZB prickle; **(B)** Epidermal cells of SZB stem; **(C)** Epidermal cell of GSZB stem; **(D)** Enlarged view of panel **(A)**; (**E–J)** Are enlargements of panel **(D)**; **(E,F)** Crystals in vacuoles; **(G)** Epidermal cells; **(H,I)** Chloroplasts; **(J)** Plasmodesmata. Scale bars: **(A–C)** = 5 μm; **(D)** = 2 μm; **(F,G)** = 1 μm; **(E,H)** = 500 nm; **(I)** = 200 nm; **(J)** = 100 nm. PEP, prickle epidermis; SEP, stem epidermis; P, parenchyma; RAZ, resembling abscission zone; V, vacuole; EC, epidermal cell; CP, chloroplast; CW, cell wall; CS, crystalline substance; EW: epicuticular waxes; SG, starch grains; PD, plasmodesmata.

By comparing the cell morphology of PZB, SZB and GSZB (Figures 5A–C), it was found that there were far more crystalline substances (CS) in the vacuoles of prickle cells than stem cells. The detail structures of these crystalline substances (CS) were shown in Figures 5E,F, and the morphology of Figure 5F maybe evolve from Figure 5E. Besides, we combined this finding with that of microstructure, and speculated that this kind of crystalline substances (CS) might be a precursor for lignin synthesis.

Figure 5G shows the epidermal cells of prickles. These epidermal cells were dead cells without any organelles, and their cell walls (CW) were thickened (Dominguez et al., 2011; Guzman et al., 2014; de la Fuente et al., 2017). Besides, there was a layer of epidermal wax (EW) on the outside of cell walls which means these cells were highly lignified (Jenks et al., 1994; Ma Z. Y. et al., 2016; Pereira, 2018).

Figures 5H,I show the chloroplasts (CP) structure of prickle cells. In addition to starch granules (SG) and thylakoids (Khan et al., 2020), a kind of dark crystalline substances (CS) were found in chloroplasts (CP), which means that during the process of cell lignification, certain changes have also occurred in chloroplasts (CP).

Figure 5J shows that there were plasmodesmata (PD) between cells in the resembling abscission zone (RAZ) (Li et al., 2012). In addition, it was found that the cells near the two sides of the resembling abscission zone (RAZ) had no obvious difference in ultrastructure.

### Ribonucleic acid sequencing, assembly and functional annotation

**FIGURE 6:**
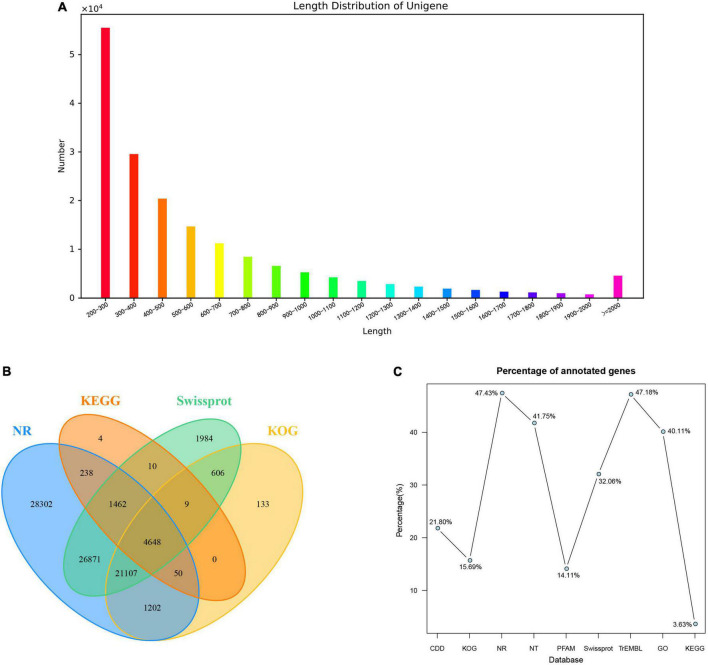
Summary of the assembly and annotations. **(A)** Length distribution of unigenes; **(B)** Venn diagram of annotation results; **(C)** Annotation ratio of different databases.

In total, 9 cDNA libraries from the young stem bark of PZB, SZB, and GSZB were constructed and sequenced. The raw reads of the libraries were deposited in the NCBI Sequence Read Archive (SRA) with Bioproject accession number: PRJNA752915 and SRA accession number: SRR15440480-SRR15440497. After quality control and low-quality data screening, clean reads were obtained and assembled into 176,862 unigenes, with an average length of 596.7 bp. Among these unigenes, the maximum length was 16,917 bp, the minimum length was 201 bp (Figure 6A).

A total of 95,931 (54.24%) unigenes were annotated using nine public databases. In summary, 38,563 (21.80%), 27,755 (15.69%), 83,880 (47.43%), 73,846 (41.75%), 24,950 (14.11%), 56,697 (32.06%), 83,435 (47.18%), 70,937 (40.11%), and 6,421 (3.63%) unigenes were annotated in the CDD, KOG, NR, NT, PFAM, Swissprot, TrEMBL, GO and KEGG databases, respectively (Figures 6B,C). According to the Nr database, 37,367 (21.13%) unigenes exhibited significantly higher homology with sequences from *Citrus sinensis* than *Citrus clementina* and other species (Supplementary Figure 2A). Unigenes annotated in the KOG database were mainly distributed in “signal transduction mechanisms” and “general function prediction only” function classes (Supplementary Figure 2B). Besides, the annotation results of unigenes in GO gene function classification and KEGG pathway classification were shown in Supplementary Figures 2C,D.

### Differentially expressed genes identification and enrichment analyses

**FIGURE 7:**
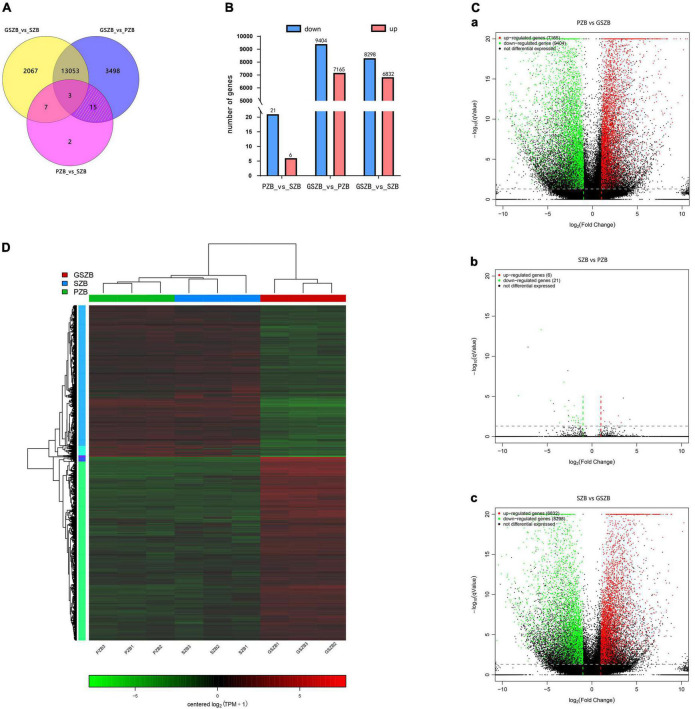
**(A)** Venn diagram of differentially expressed genes (DEGs) among two or three comparisons; **(B)** Total number of upregulated and downregulated DEGs between different groups; **(C)** Volcano plot of DEGs for PZB_vs_GSZB, SZB_vs_PZB and SZB_vs_GSZB; **(D)** Heatmap of DEGs based on hierarchical clustering analysis.

The TPM values (Transcripts Per Million) were calculated for each unigene. Besides, | log_2_(fold change)| > 2 and *q*-value < 0.05 were set as thresholds for significant DEGs selection. It can be seen from Figures 7A-C that a total of 27 DEGs were detected between PZB and SZB libraries (6 upregulated and 21 downregulated genes); 16,569 DEGs were detected between GSZB and PZB libraries (7,165 upregulated and 9,404 downregulated genes); 15,130 DEGs were detected between GSZB and SZB libraries (6,832 upregulated and 8,298 downregulated genes) (Supplementary Tables 4–6).

In order to screen for genes that affect prickle development, the DEGs shared by PZB_vs_SZB and PZB_vs_GSZB were selected, and the DEGs of SZB_vs_GSZB were excluded. As a result, 15 ungenes were selected as candidate genes (white slashed area in Figure 7A and Supplementary Table 3), and quantitative real-time PCR analysis was performed on them. The results of qRT-PCR and RNA-Seq were found to be in close agreement, validating the accuracy of sequencing (Supplementary Figure 3).

The hierarchical clustering results of DEGs in PZB, SZB, and GSZB groups are shown in Figure 7D. Green and red indicate high and low expression levels, respectively. From it can be seen that different groups of the same varieties can be clustered together, indicating that the data obtained were reliable and reproducible.

The significant DEGs from different groups were functionally categorized using GO (Gene Ontology) enrichment analyses (Supplementary Figures 4A–C and Supplementary Tables 7–9). The results found that the significant DEGs between the PZB and SZB libraries were mainly enriched in “regulation of shoot apical meristem development (GO:1902183),” “regulation of flower development (GO:0009909),” “specification of axis polarity (GO:0065001),” “polarity specification of adaxial/abaxial axis (GO:0009944),” “xyloglucosyl transferase activity (GO:0016762),” “xyloglucan metabolic process (GO:0010411),” and “plant hormone signal transduction (GO:0009739)” (Supplementary Figure 4D and Supplementary Table 10). In addition, the significant DEGs between SZB and GSZB libraries as well as between PZB and GSZB libraries are shown in Supplementary Figures 4E,F and Supplementary Tables 11, 12. To better display the results, the top 30 GO terms with the highest enrichment between PZB and SZB were selected to draw directed acyclic graphs (Supplementary Figure 5). The results of DAGs contain three parts: biological process, cellular component and molecular function, which are shown in Supplementary Figures 5A–C, respectively.

### Identification of unigenes related to prickle development

**FIGURE 8:**
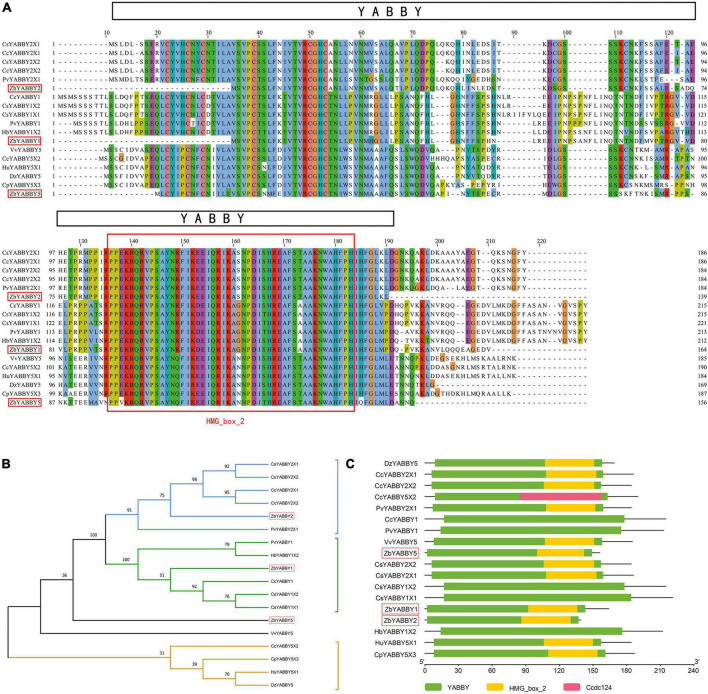
Phylogenetic analysis of ZbYABBYs and its homologous proteins. **(A)** Multiple alignments of deduced amino acid sequences of ZbYABBY2, ZbYABBY1, and ZbYABBY5 proteins with other functionally characterized YABBYs; **(B)** Phylogenetic analyses of ZbYABBY2, ZbYABBY1, and ZbYABBY5 in other plants; **(C)** Conserved domain analysis of ZbYABBY2, ZbYABBY1, and ZbYABBY5.

In this section, detailed analyses of 15 candidate genes affecting the development of *Zanthoxylum bungeanum* prickles were carried out (*ZbYABBY2*, *ZbYABBY1*, *ZbYABBY5*, *ZbWRKY28*, *ZbAZG2*, *ZbLOG5*, *ZbIAA33*, *ZbGh16*, *ZbGh16X1*, *Zb19224c0g1*, *Zb33022c0g3*, *Zb36195c1g2*, *Zb51123c2g3*, *Zb40454c0g1*, and *Zb22746c0g1*) (Supplementary Table 3). Among them, the expression of *ZbYABBY2*, *ZbYABBY1*, *ZbYABBY5*, and *ZbWRKY28* were higher at PZB than at SZB and GSZB. The phylogenetic analysis of ZbYABBYs and its homologous proteins was shown in Figure 8. It can be found that ZbYABBYs was first clustered with CsYABBYs and CcYABBYs, and its conserved domain was HMG_box_2 (Siegfried et al., 1999; Bartholmes et al., 2012).

Bowman’s research found that the YABBY gene family of *Arabidopsis thaliana* comprises six members that probably encode transcriptional regulators. Each member of the family is expressed in a polar manner in one or more above-ground lateral organs, and in every lateral organ at least one family member is expressed (Bowman, 2000). Besides, previous studies have shown that YABBYs are related to the establishment of polarity in angiosperm lateral organs (Bowman et al., 2002).

The phylogenetic analysis of ZbWRKY28 and its homologous proteins was shown in Supplementary Figure 6. The transcription factors of WRKY class have been proved to control trichome, seed coat, and root hair development in *Arabidopsis thaliana* (Eulgem et al., 2000; Johnson et al., 2002). Glandular trichome formation in *Gossypium* spp. and *Rosa chinensis* is also regulated by them (Ma D. et al., 2016; Hibrand Saint-Oyant et al., 2018). Therefore, *ZbYABBY2*, *ZbYABBY1*, *ZbYABBY5*, and *ZbWRKY28* may affect the development of *Zanthoxylum bungeanum* prickles.

The candidate genes of *ZbAZG2*, *ZbLOG5*, and *ZbIAA33* were related to plant hormone signal transduction. The results of multiple alignments, phylogenetic analyses and conserved domain analysis of their deduced amino acid sequences are respectively shown in Supplementary Figures 7–9. AZG1 and AZG2 proteins were shown to function as cytokinin transporters which can opening doors for cytokinin trafficking at the ER membrane (Romanov and Schmulling, 2021). Besides, AZGs were found can regulate lateral root emergence in *Arabidopsis thaliana* (Tessi et al., 2021). Kuroha’s research suggested that LOGs (LOG1-LOG9) played a pivotal role in regulating cytokinin activity and worked in the direct activation pathway in *Oryza sativa* shoot meristems (Kuroha et al., 2009; Tokunaga et al., 2012). Lv and his team found that IAA33 overexpression enhanced root distal stem cell differentiation (Audran-Delalande et al., 2012; Lv et al., 2020). In our research, the expression levels of *ZbAZG2*, *ZbIAA33*, and *ZbLOG5* in PZB were significantly higher than in SZB and GSZB. Combined with previous studies, it is speculated that the development of *Zanthoxylum bungeanum* prickles is regulated by plant hormones.

The phylogenetic analysis of ZbGh16Xs and its homologous proteins was shown in Supplementary Figure 10. Conserved domain analysis of ZbGh16X1 and ZbGh16 indicated that ZbGh16Xs possess a Glyco_hydro_16 central domain and an XET_C C-terminal domain. The activity of the XET_C domain is characterized by endolytic cleavage of the xyloglucan chain, which transfers the reducing end (donor) of the chain to the non-reducing end (acceptor) of a different xyloglucan chain (Atkinson et al., 2009).

Besides, the Gh16 (glycosyl hydrolase 16) family contains multiple glucanases and endo-acting galactanases (Strohmeier et al., 2004), which are involved in the Endotrans-glucosylation process. The process of endotrans-glucosylation allows cell expansion by temporarily loosening the cell wall in rapidly growing cells, and incorporates newly synthesized xyloglucan chains into the cell wall for reorganization in plants (Nishitani and Tominaga, 1992). Therefore, ZbGh16X1 and ZbGh16 may regulate the process of cell wall thickening and cell lignification. In our study, it was found that the expression levels of ZbGh16X1 and ZbGh16 in PZB were much higher than those in SZB and GSZB. This result explained exactly why the prickle cells had a higher degree of lignification than stem cells (Figures 4, 5).

### Metabolomic profiling

**FIGURE 9:**
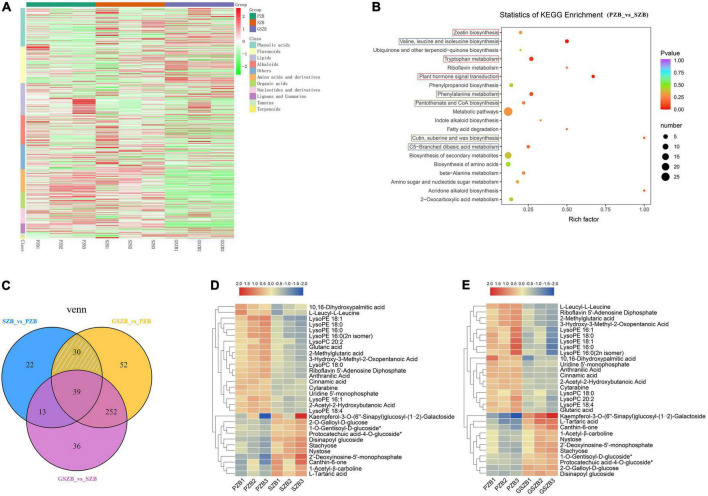
Metabolomic profiling results. **(A)** Heatmap of differentially accumulated metabolites (DAMs) based on hierarchical clustering analysis; **(B)** KEGG enrichment results between SZB and PZB. Abscissa represents Rich factor; Ordinate represents the name of pathways. The color of dots represents *P*-value; The size of dots represents the number of DAMs; **(C)** Venn diagram of DAMs among two or three comparisons; **(D)** Heatmap of 30 DAMs between SZB and PZB; **(E)** Heatmap of 30 DAMs between GSZB and PZB.

A widely targeted metabolomic analysis was performed to produce a metabolic profile. The total ion current (TIC) of all the samples showed high stability, large peak capacity, and good retention time, indicating the reliability and accuracy of the data (Supplementary Figure 11). Besides, a total of 802 metabolites were identified in the young stem bark of *Zanthoxylum bungeanum* and were divided into 11 categories, primarily lipids, phenolic acids, lignans and coumarins, just as Figure 9A shows (Supplementary Table 13).

### Identification of differentially accumulated metabolites

The DAMs of each comparison group were selected by setting VIP ≥ 1 and fold change ≥ 2 or ≤ 0.5 as thresholds. As a results, 373, 340, and 104 DAMs were detected between GSZB_vs_PZB, GSZB_vs_SZB, and SZB_vs_PZB, respectively (Supplementary Tables 14–16 and Supplementary Figures 12A–C).

In order to screen for metabolites that affect prickle development, the DAMs shared by PZB_vs_SZB and PZB_vs_GSZB were selected, and the DAMs of SZB_vs_GSZB were excluded. Finally, 30 metabolites were selected as candidate metabolites (white slashed area in Figure 9C). Among them, the content of metabolites such as LysoPE, LysoPC, ainnamic acid, and 10,16-dihydroxypalmitic acid in PZB variety was higher than that in SZB and GSZB varieties. Besides, the content of metabolites such as stachyose, nystose, 2′-Deoxyinosine-5′-monophosphate, glutaric acid and Protocatechuic acid-4-O-glucoside in PZB variety was lower than that in SZB and GSZB varieties (Figures 9D,E).

The 10,16-dihydroxypalmitic acid is a precursor for the synthesis of cutin (Kolattukudy and Walton, 1972; Croteau and Kolattukudy, 1974), and its content in PZB was significantly higher than that in GSZB and SZB, indicating that prickle cells were lignified earlier than stem cells, which fits well with the results observed in transmission electron microscope (Figures 5A–C). In addition, Gao’s research found that increased levels of the lipid metabolites such as lysoPC and lysoPE can help *Arabidopsis thaliana* resist extreme conditions (Wang et al., 2006; Gao et al., 2010). This phenomenon also found in *Zanthoxylum bungeanum*, which tends to grow more prickles under extreme drought conditions to protect themselves. And this may be the reason for why the content of lysoPC and lysoPE was higher in PZB than in SZB and GSZB varieties.

### Enrichment analysis of the differentially accumulated metabolites

The KEGG pathway enrichment analysis of DAMs from each comparison group showed that they were enriched in multiple metabolic pathways, including cutin, suberine and wax biosynthesis; plant hormone signal transduction; zeatin biosynthesis; valine, leucine and isoleucine biosynthesis; etc. (Figure 9B and Supplementary Figures 12D–F).

**FIGURE 10:**
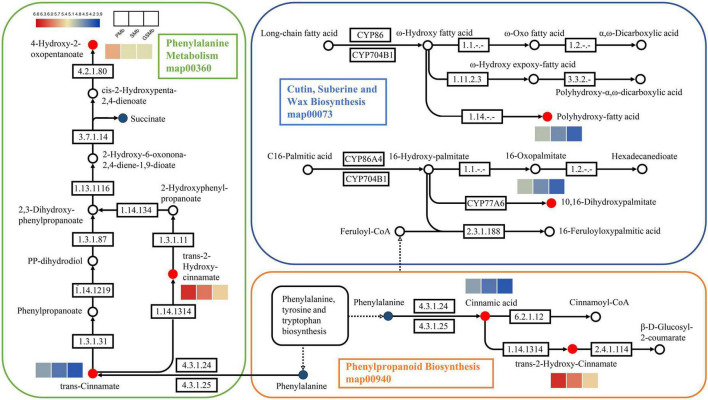
Differentially accumulated metabolites (DAMs) in the biosynthetic pathway of young stem bark of *Zanthoxylum bungeanum*. [KEGG map: The red dots represent up-regulation, the green dots represent down-regulation, and the blue dots represent no difference. Heatmap: The color scale from blue (low) to red (high) represents the fold change values].

Among them, the pathways of map00360, map00073, and map00940 were related to the biosynthesis of cutin, suberine and wax (Figure 10). And it can be found that the content of DAMs, such as 4-hydroxy-2-oxopentanoate, trans-2-hydroxy-cinnamate, trans-cinnamate, polyhydroxy-fatty acid, 10,16-dihydroxypalmitate, cinnamic acid, was significantly higher in PZB variety than in SZB and GSZB varieties. In addition, the pathways of map00380, map04075 and map00908 were related to plant hormone signal transduction, while the pathways of map0066 and map00290 were related to the biosynthesis of valine, leucine, and isoleucine. Their results were shown in Supplementary Figures 13, 14 respectively.

### Correlation analysis between transcriptome and metabolome data

The accumulation of metabolites is controlled by many exogenous and endogenous factors. Therefore, metabolomics and transcriptomics need to be correlated to identify functional genes and metabolic pathways.

**FIGURE 11:**
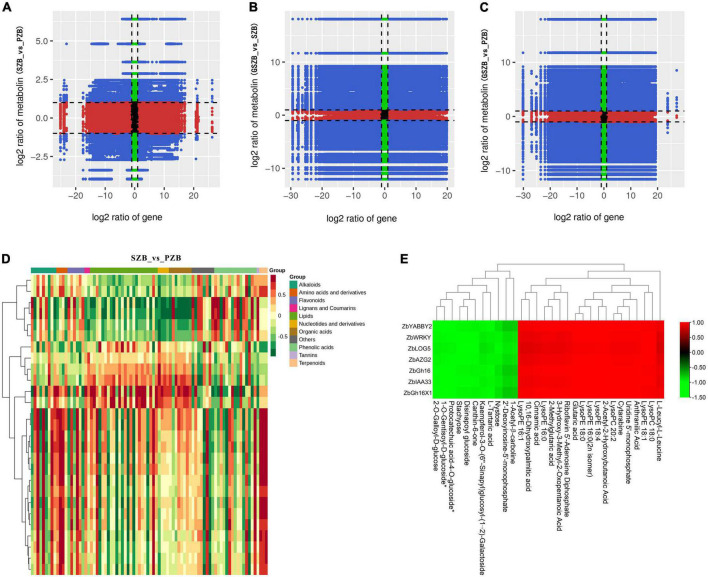
**(A–C)** Nine-quadrant diagram of correlations of differentially expressed genes (DEGs) and differentially accumulated metabolites (DAMs); (From left to right, top to bottom, are 1 to 9 quadrants respectively); **(D)** The correlation heatmap of DEGs and DAMs between SZB and PZB libraries; **(E)** The clustering heatmap of candidate DEGs and candidate DAMs.

The relationship between DEGs and DAMs with a PCC (Pearson correlation coefficient) ≥ 0.8 was displayed in nine-quadrant graphs (Figures 11A–C). As well as, the correlation heatmap of DEGs and DAMs between SZB and PZB libraries was shown in Figure 11D (The similar results of GSZB_vs_SZB and GSZB_vs_PZB were shown in Supplementary Figures 15, 16).

For the 7 candidate DEGs and the 30 candidate DAMs, a separate clustering heatmap was drawn to intuitively reflect the relationship between them (Figure 11E). The genes (*ZbYABBY2*, *ZbWRKY*, *ZbLOG5*, *ZbAZG2*, *ZbGh16*, *ZbIAA33*, and *ZbGh16X1*) were positively correlated with metabolites such as LysoPE, LysoPC, 10,16-dihydroxypalmitic acid, and negatively correlated with metabolites such as stachyose, nystose, canthin-6-one.

## Discussion

This study was designed to uncover the morphological characteristics and molecular mechanism of prickle development of *Zanthoxylum bungeanum*. To this end, the tissues of “Wild Prickly *Zanthoxylum bungeanum* (PZB),” “Wild Smooth *Zanthoxylum bungeanum* (SZB),” and “Grafted Smooth *Zanthoxylum bungeanum* (GSZB)” were collected for experiments. And the techniques of Fourier-transform infrared spectroscopy (FTIR), optical microscopy, transmission electron microscopy (TEM), transcriptomic, quantitative real-time PCR (qRT-PCR), and metabolomic and multi-omics analysis were used for investigation.

Fourier-transform infrared spectroscopy (FTIR) results revealed that the absorption spectra of prickles and stems were different. Prickles had band at 1617 and 1110 cm^–1^, while stems had strong band at 3319 and 1999 cm^–1^. According to the band correspondence, the prickles of *Zanthoxylum bungeanum* contain unique aromatic compounds (Breub et al., 2010; Lupoi et al., 2015; Reyes-Rivera and Terrazas, 2017), while the stems contain compounds with -NH_2_ or -NH bonds.

The results of morphological studies showed that the prickles of *Zanthoxylum bungeanum* had no vascular bundle. The growth directions of prickle and stem cells were at a vertical angle, and there was a resembling abscission zone (RAZ) between them. The existence of RAZ explains well why prickles tend to peel off the stems. Besides, prickles lignified earlier than stems, and the deposits in vacuoles of prickle cells were obviously more than stem cells. Combining with transcriptome, metabolome and FTIR results, these deposits may be compounds related to xyloglucan metabolism (Nishitani and Tominaga, 1992; Strohmeier et al., 2004; Atkinson et al., 2009) and cutin synthesis (Kolattukudy and Walton, 1972; Croteau and Kolattukudy, 1974; Wang et al., 2006; Gao et al., 2010).

Through transcriptomic analysis and qRT-PCR validation, 9 DEGs (*ZbYABBY2*, *ZbYABBY1*, *ZbYABBY5*, *ZbWRKY*, *ZbLOG5*, *ZbAZG2*, *ZbGh16*, *ZbIAA33*, and *ZbGh16X1*) related to prickle development were screened and validated. Their functional annotation, phylogenetic analyses, multiple sequence alignment, and conserved domain analyses revealed that *ZbYABBY1*, *ZbYABBY2*, *ZbYABBY5*, and *ZbWRKY* were involved in the shoot apical meristem development and the flower development (Siegfried et al., 1999; Bowman, 2000; Eulgem et al., 2000; Bowman et al., 2002; Johnson et al., 2002; Bartholmes et al., 2012; Ma D. et al., 2016; Hibrand Saint-Oyant et al., 2018); *ZbLOG5*, *ZbAZG2*, and *ZbIAA33* were involved in the plant hormone signal transduction and the response to gibberellin (Kuroha et al., 2009; Audran-Delalande et al., 2012; Tokunaga et al., 2012; Lv et al., 2020; Romanov and Schmulling, 2021; Tessi et al., 2021); *ZbGh16* and *ZbGh16X1* were involved in the cutin, suberin and wax biosynthesis (Nishitani and Tominaga, 1992; Strohmeier et al., 2004; Atkinson et al., 2009).

Metabolomic analysis identified 802 compounds, and screened out 30 candidate metabolites related to the development of prickles. The metabolites such as LysoPE, LysoPC and 10,16-dihydroxypalmitic were related to the biosynthesis of cutin, suberine and wax; The metabolites such as Indole-3-acetate, N6-(delta2-isopentenyl)-adenine and tryptamine were related to plant hormone signal transduction; The metabolites such as (S)-2-acetolactate, (R)-3-hydroxy-3-methyl-2-oxopentanoate and (S)-citramalate were related to valine, leucine and isoleucine biosynthesis (Kolattukudy and Walton, 1972; Croteau and Kolattukudy, 1974; Wang et al., 2006; Gao et al., 2010). In addition, through multi-omics analysis, DEGs and DAMs with PCC (Pearson correlation coefficient) ≥ 0.8 were screened out.

This study elucidated the developmental mechanism of prickles at morphological and molecular levels. The candidate genes and metabolites that affected prickle development were screened out, and the morphological characteristics of prickles were observed in detail. In conclusion, this study filled the gap in the research field of *Zanthoxylum bungeanum* prickles and provided a theoretical basis for the breeding of non-prickle *Zanthoxylum bungeanum*.

## Data availability statement

The original contributions presented in this study are publicly available. This data can be found here: NCBI, PRJNA752915.

## Author contributions

SL and KS conceived and designed the study. SL, KS, JS, BS, JH, and TZ participated in the coordination of the study. KS performed the experimental measurements, processed the experimental data, interpreted the data, and drafted and revised the manuscript. SL, KS, and JS reviewed and revised the manuscript. All authors contributed to the article and approved the submitted version.

## Footnotes

1
https://www.ncbi.nlm.nih.gov/Structure/bwrpsb/bwrpsb.cgi
