# Supplementary material for: Combined morphological and multi-omics analyses to reveal the developmental mechanism of Zanthoxylum bungeanum prickles
Source: Front Plant Sci. 2022 Aug 22;13:950084. doi: 10.3389/fpls.2022.950084 (PMC9441855; doi:10.3389/fpls.2022.950084)
Supplement: Supplementary file 2 [file Data_Sheet_2.pdf]

Supplementary

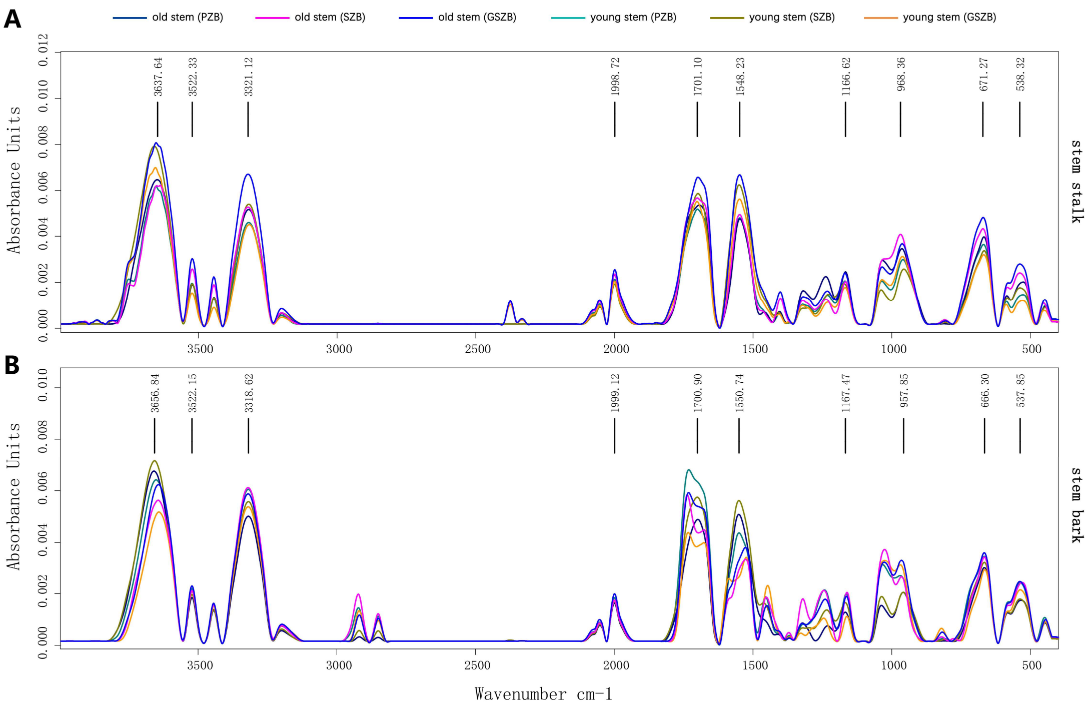

Fig. S1. FTIR Absorption Spectra of Stalk and Bark. (A) The absorption spectrum of young and old stalk of PZB, SZB and GSZB; (B) the absorption spectrum of young and old bark of PZB, SZB and GSZB.

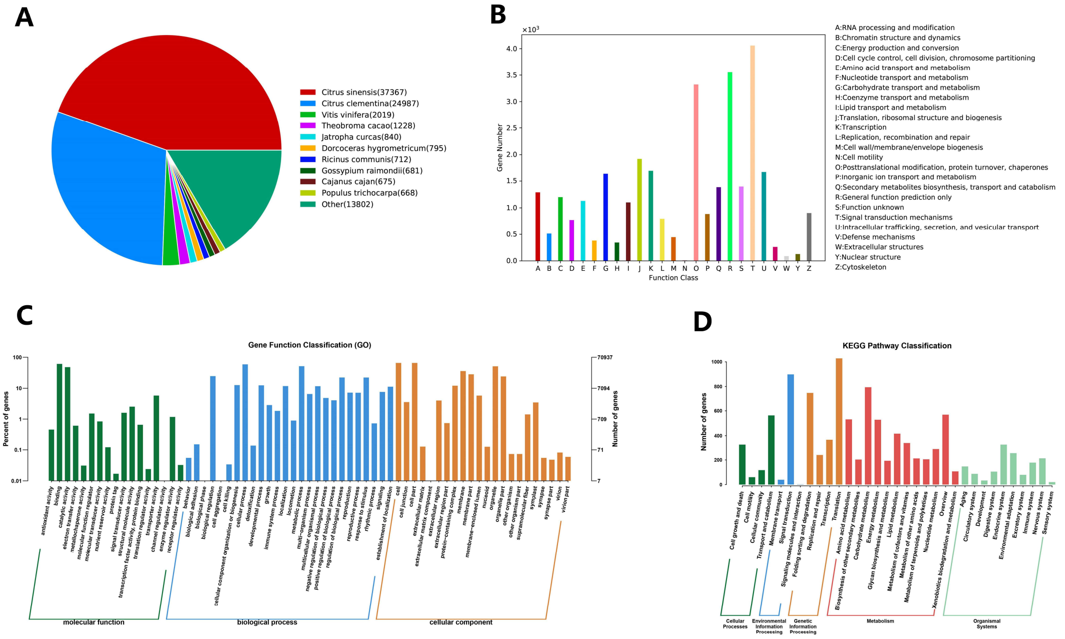

Fig. S2. Visualization of Functional Annotations. (A) Annotation results of NR database; (B) Annotation results of KOG database; (C) Annotation results of the GO database; (D) Annotation results of the KEGG database.

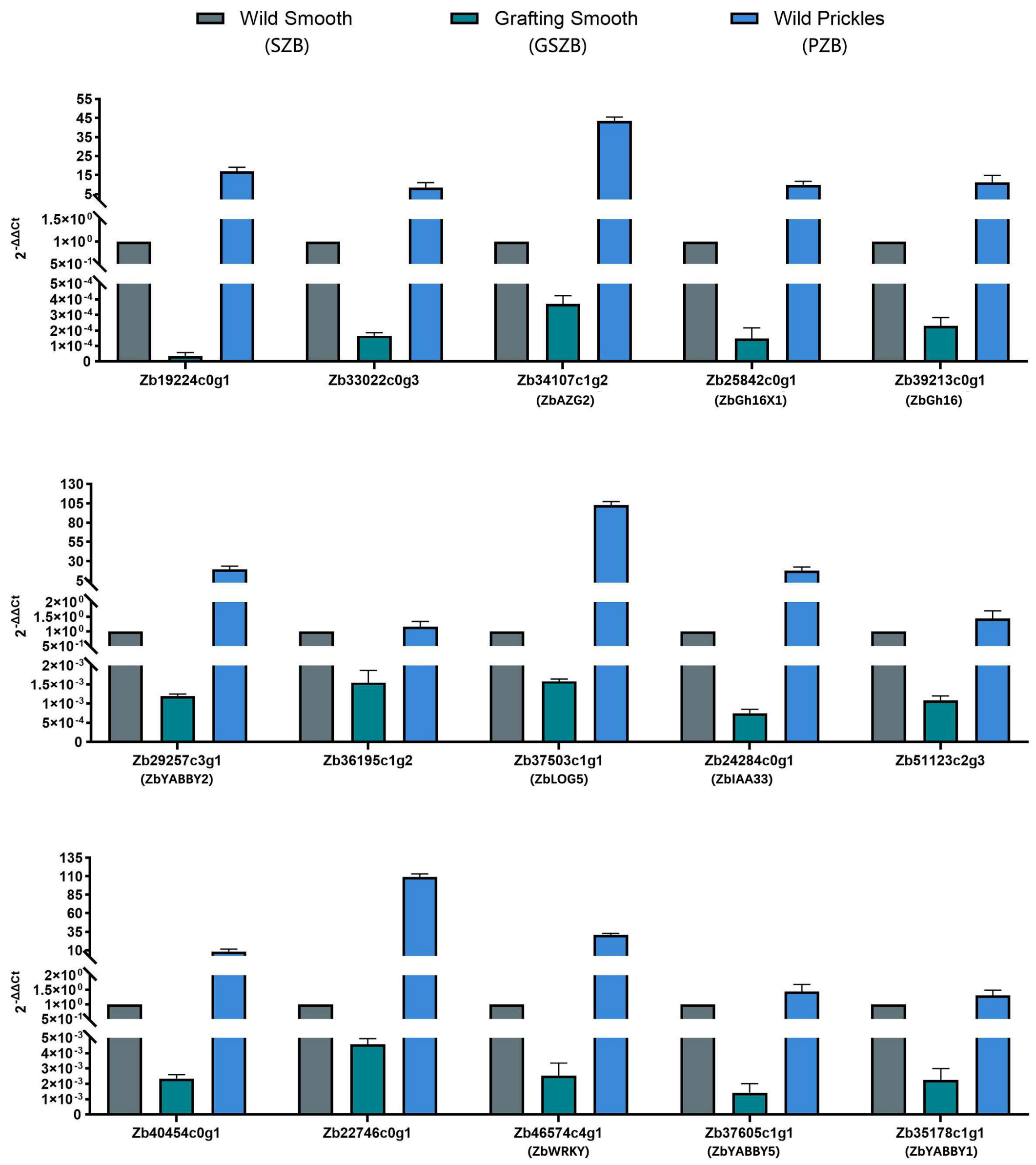

Fig. S3. Quantitative real-time PCR Results of Candidate Genes. **Note**: Error bars represent standard deviation of three replicates.

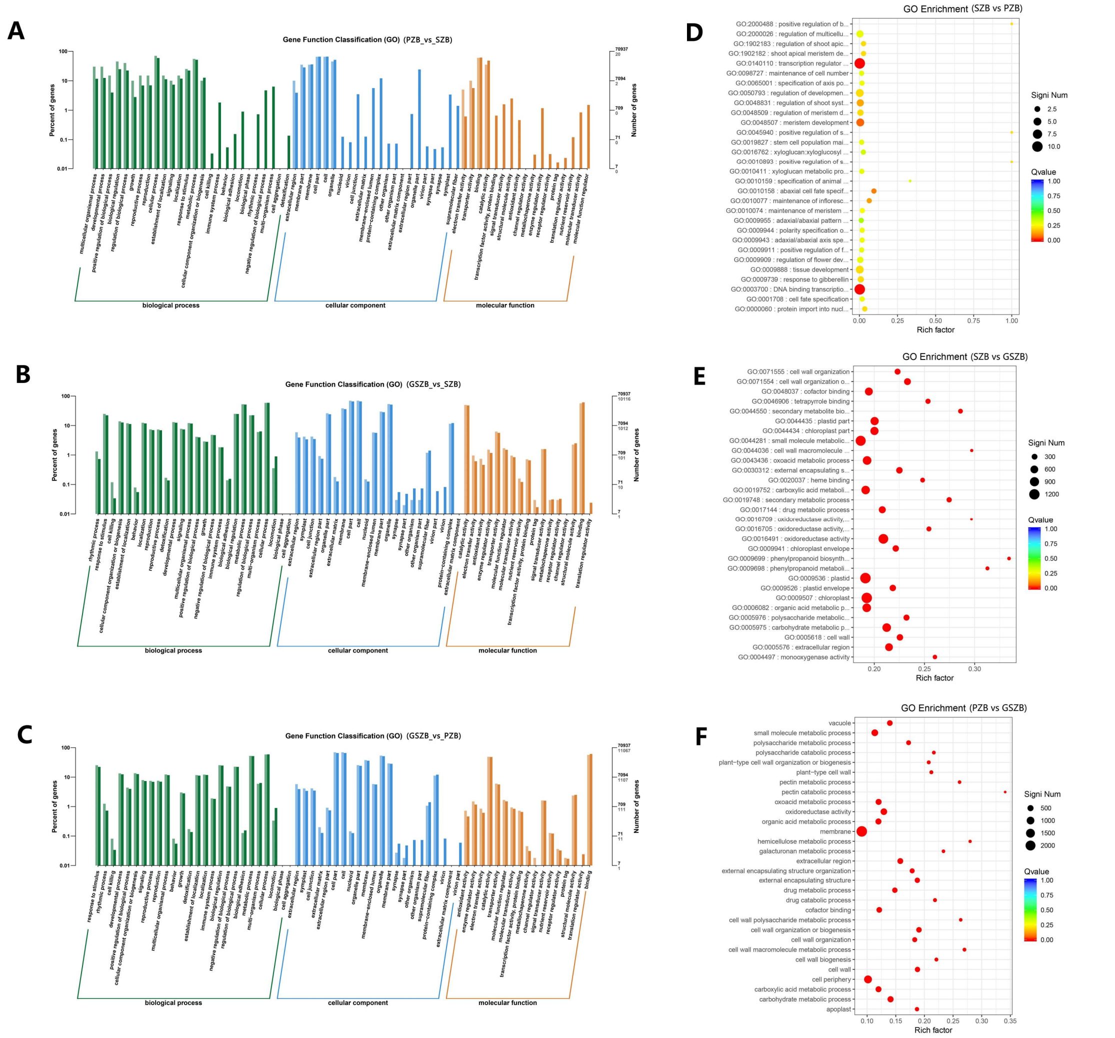

Fig. S4. GO Analysis Results. (A) GO annotation classification of DEGs between PZB and SZB; (B) GO annotation classification of DEGs between SZB and GSZB; (C) GO annotation classification of DEGs between PZB and GSZB; (D) Enrichment results of DEGs between PZB and SZB ; (E) Enrichment results of DEGs between SZB and GSZB; (F) Enrichment results of DEGs between PZB and GSZB.

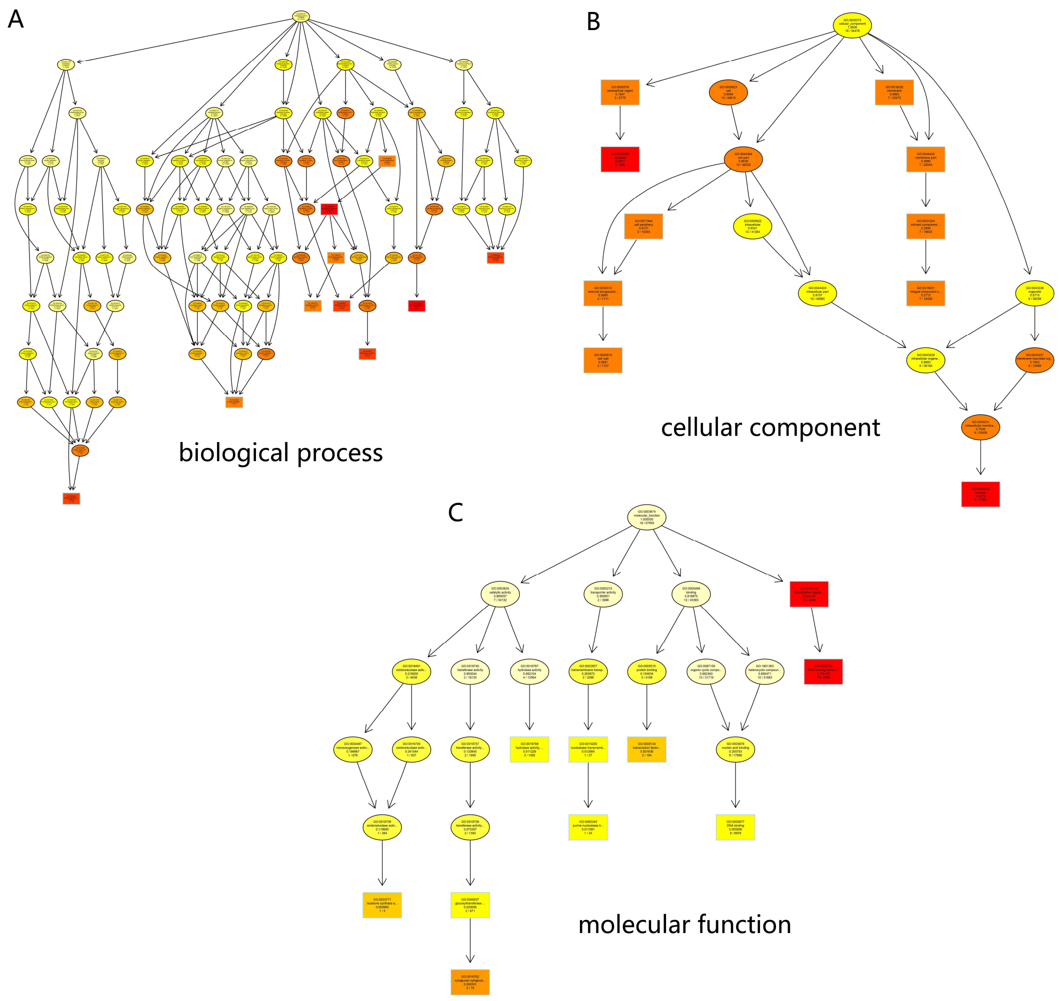

Fig. S5. Directed Acyclic Graphs between PZB and SZB. (A) DAG of Biological Process; (B) DAG of Cellular Component; (C) DAG of Molecular Function. (**Note**: Each box represents a GO term. Besides, the depth of the color represents the degree of enrichment.)

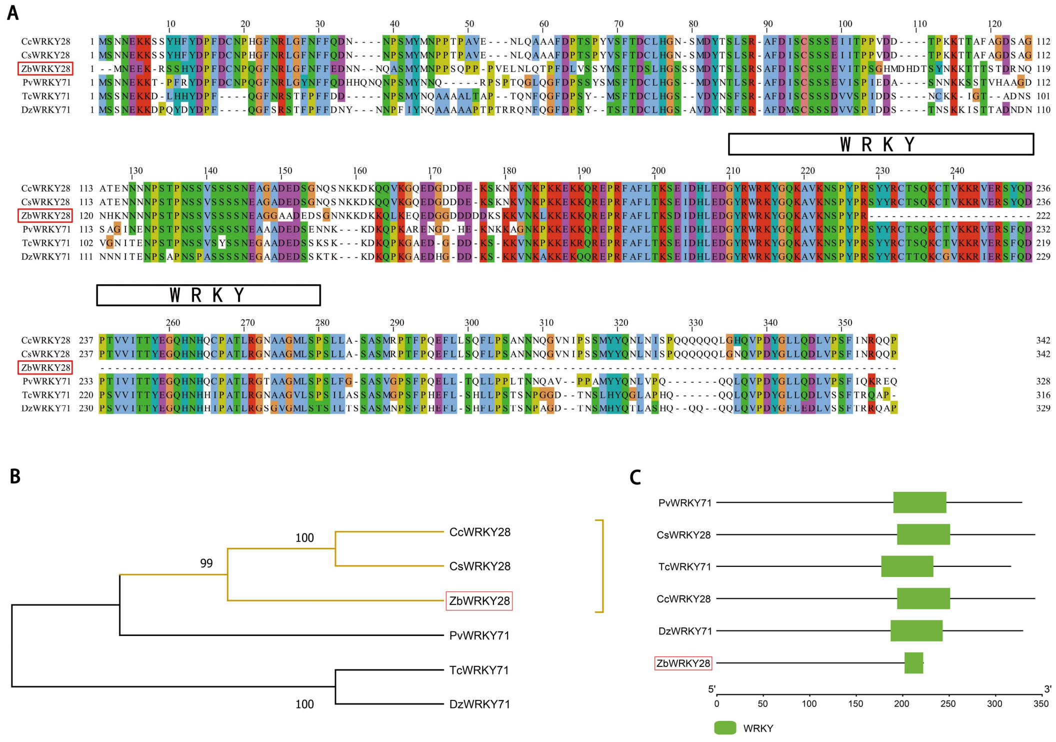

Fig. S6. Phylogenetic Analysis of ZbWRKY28 and Its Homologous Proteins. (A) Multiple alignments of deduced amino acid sequences of ZbWRKY28 proteins with other functionally characterized WRKYs; (B) Phylogenetic analyses of ZbWRKY28 in other plants; (C) Conserved domain analysis of ZbWRKY28.

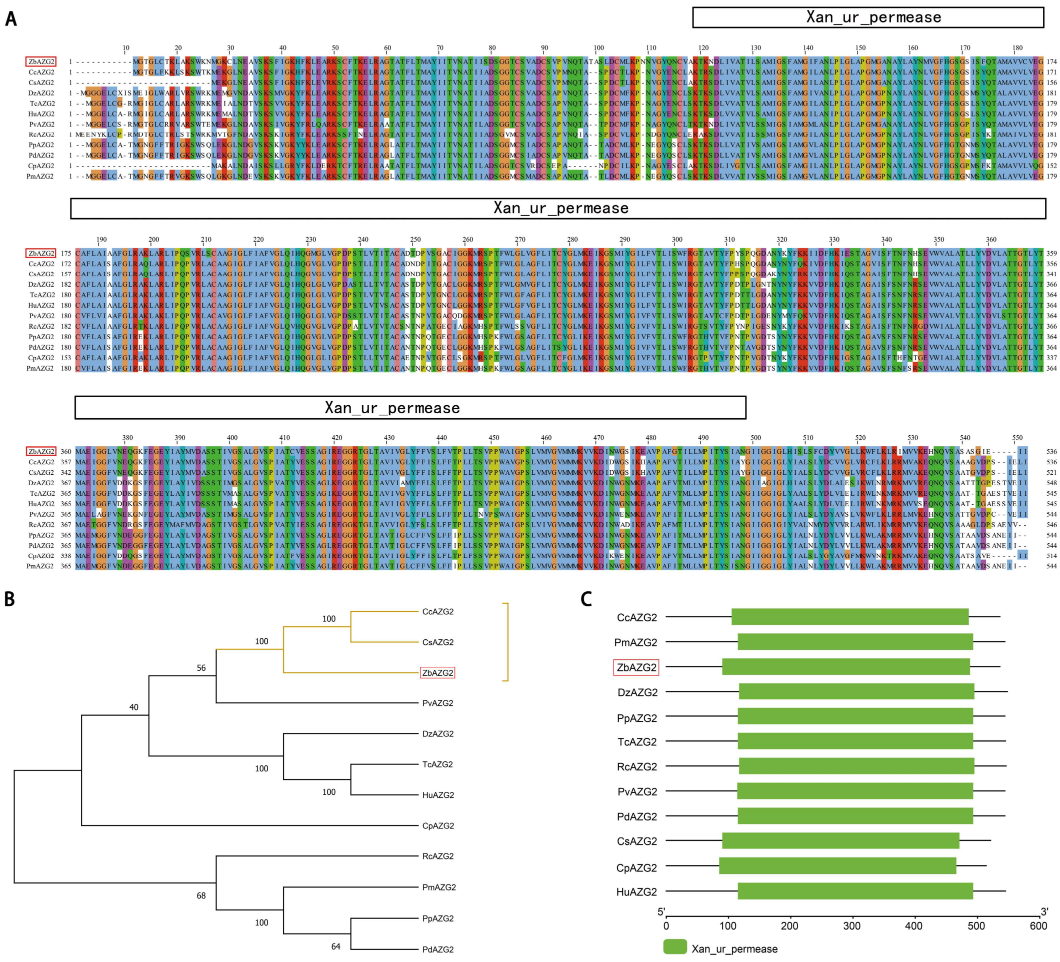

Fig. S7. Phylogenetic Analysis of ZbAZG2 and Its Homologous Proteins. (A) Multiple alignments of deduced amino acid sequences of ZbAZG2 proteins with other functionally characterized AZGs; (B) Phylogenetic analyses of ZbAZG2 in other plants; (C) Conserved domain analysis of ZbAZG2.

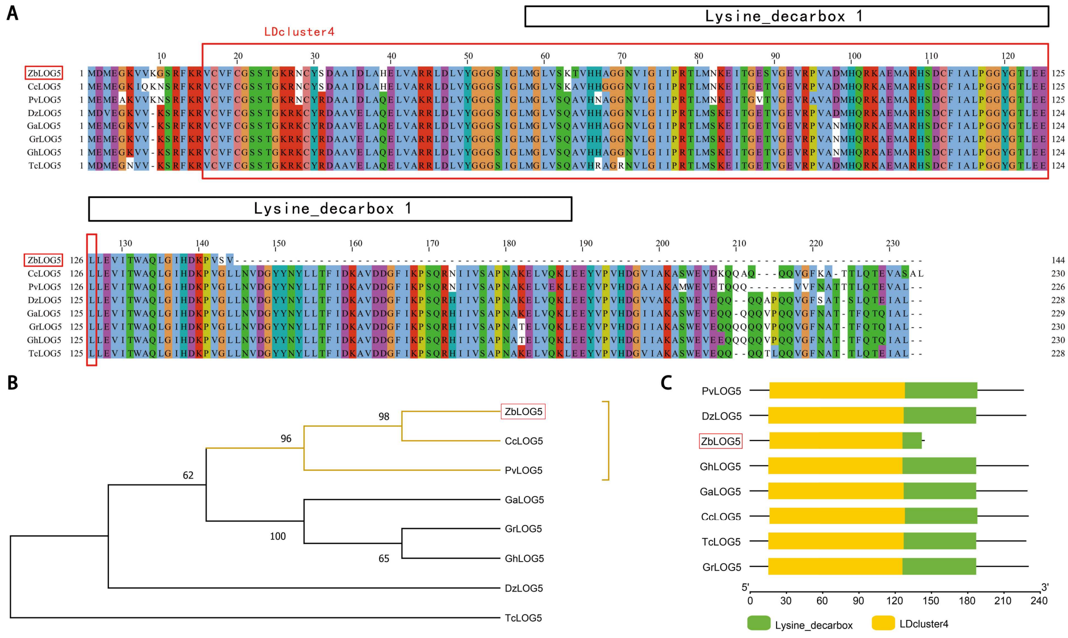

Fig. S8. Phylogenetic Analysis of ZbLOG5 and Its Homologous Proteins. (A) Multiple alignments of deduced amino acid sequences of ZbLOG5 proteins with other functionally characterized LOGs; (B) Phylogenetic analyses of ZbLOG5 in other plants; (C) Conserved domain analysis of ZbLOG5.

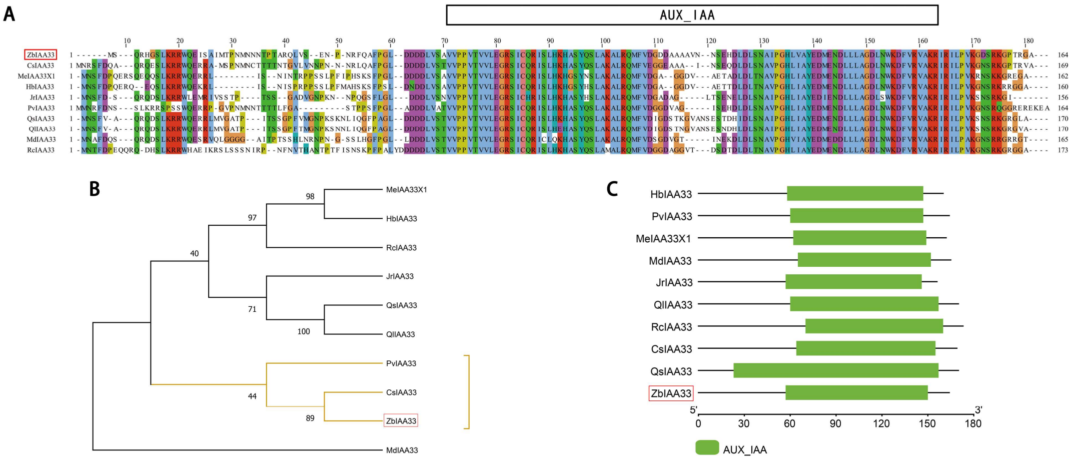

Fig. S9. Phylogenetic Analysis of ZbIAA33 and Its Homologous Proteins. (A) Multiple alignments of deduced amino acid sequences of ZbIAA33 proteins with other functionally characterized IAA33s; (B) Phylogenetic analyses of ZbIAA33 in other plants; (C) Conserved domain analysis of ZbIAA33.

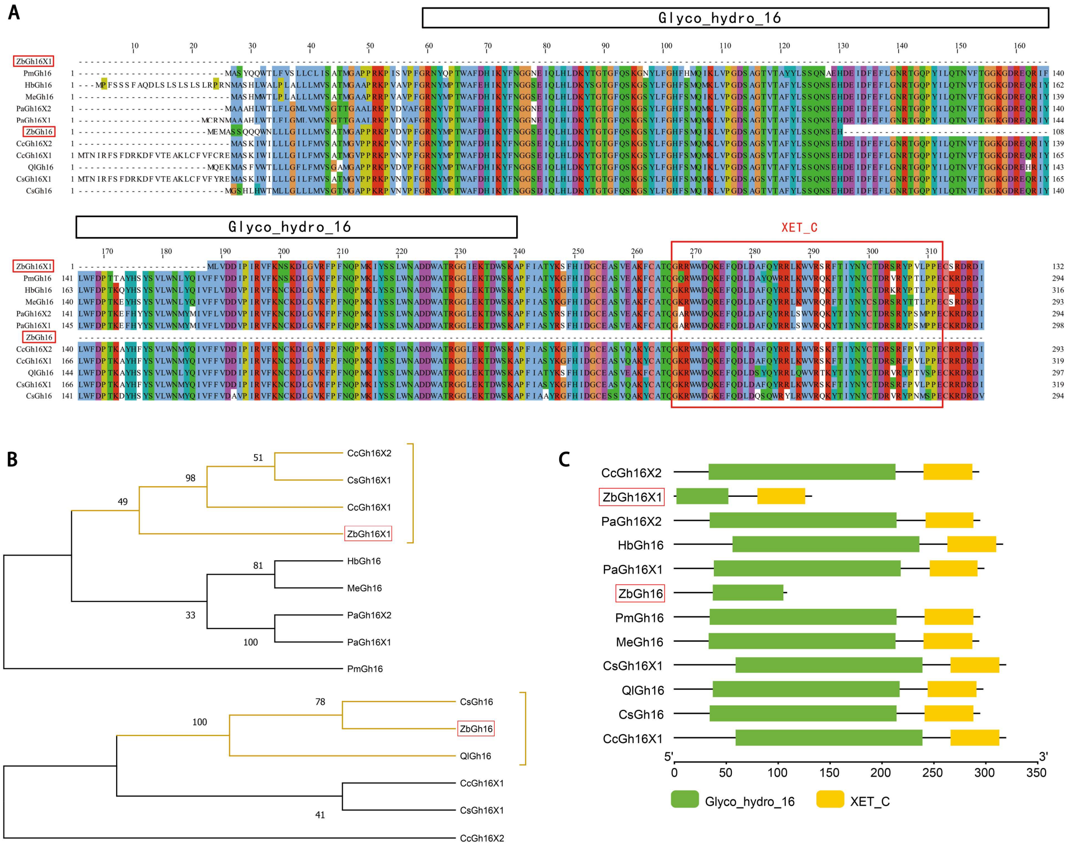

Fig. S10. Phylogenetic Analysis of ZbGh16Xs and Its Homologous Proteins. (A) Multiple alignments of deduced amino acid sequences of ZbGh16x1 and ZbGh16 proteins with other functionally characterized Gh16Xs; (B) Phylogenetic analyses of ZbGh16x1 and ZbGh16 in other plants; (C) Conserved domain analysis of ZbGh16x1 and ZbGh16.

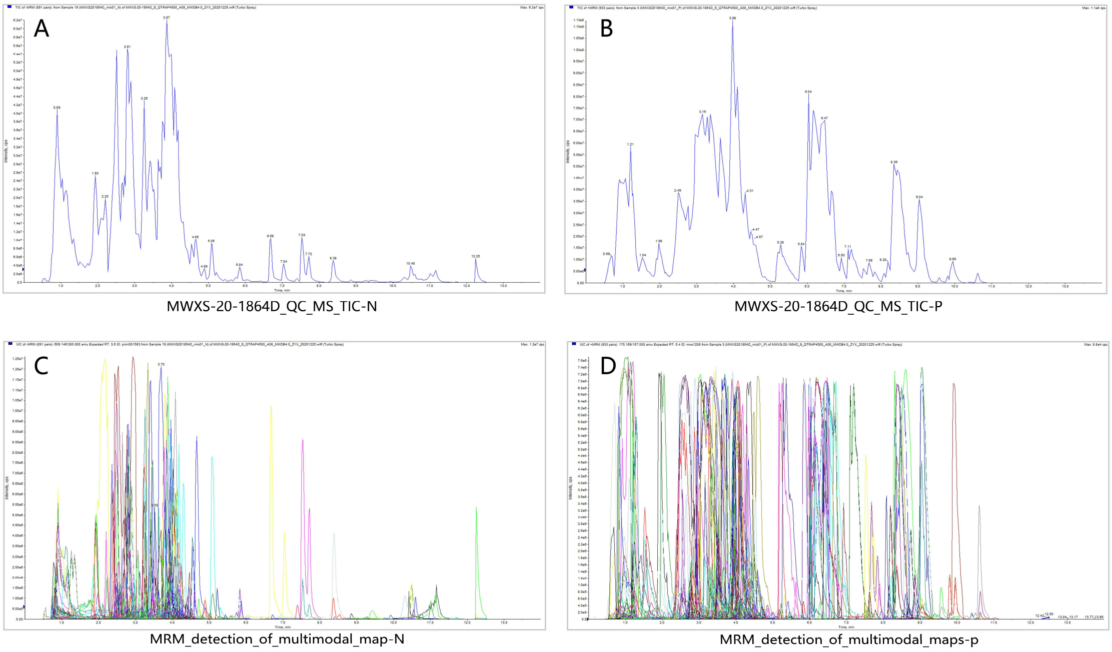

Fig. S11. Results of TIC and MRM. (A) Mass spectrometry total ion chromatogram (N stands for negative ion mode); (B) Mass spectrometry total ion chromatogram (P stands for positive ion mode); (C) MRM metabolite detection multimodal graph (N stands for positive ion mode); (D) MRM metabolite detection multimodal graph (P stands for positive ion mode).

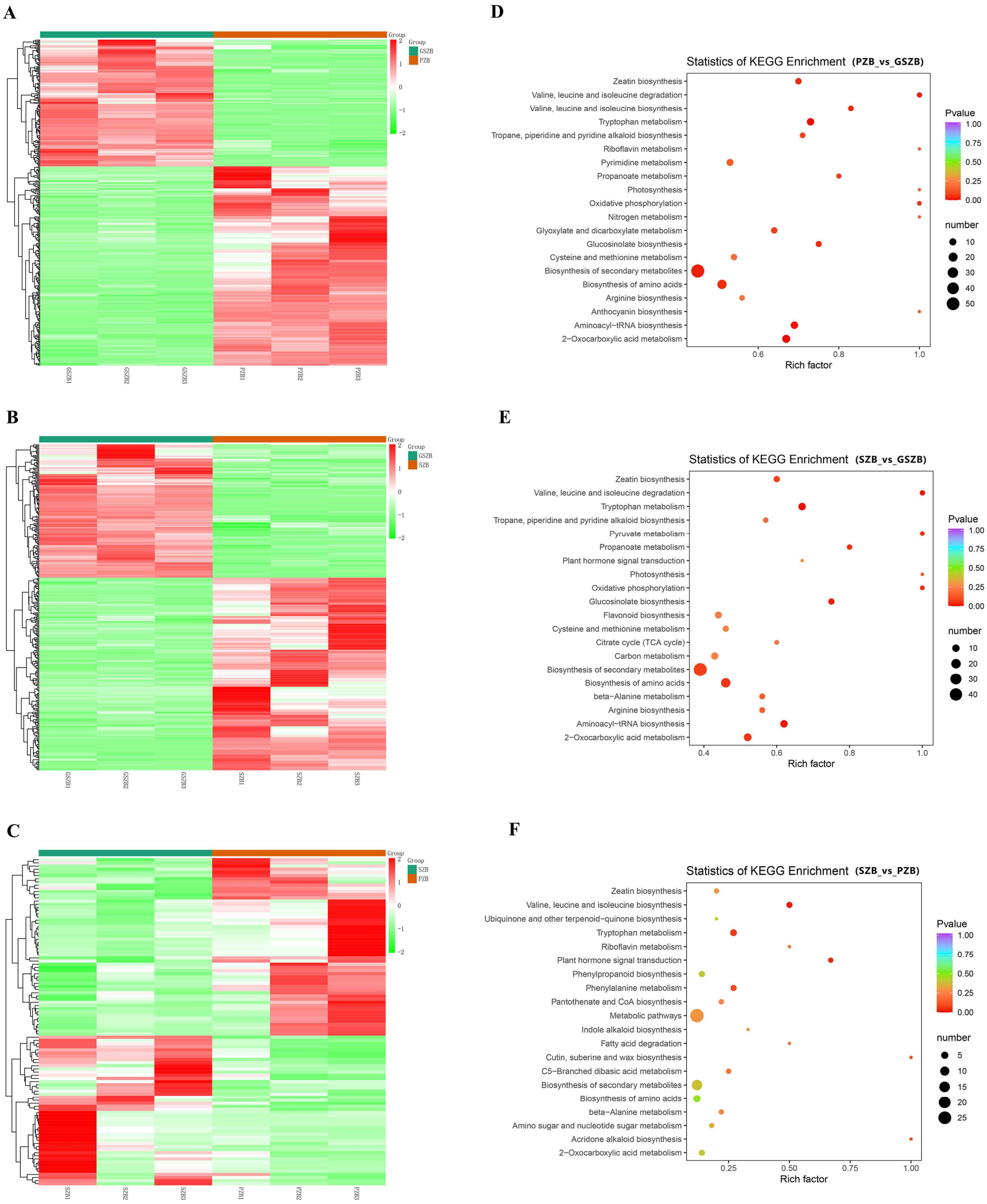

Fig. S12. Clustering Heatmap and KEGG Enrichment Results. (A) Clustering heatmap results between PZB and GSZB; (B) Clustering heatmap results between SZB and GSZB; (C) Clustering heatmap results between SZB and PZB; (D) KEGG enrichment results between PZB and GSZB; (E) KEGG enrichment results between SZB and GSZB; (F) KEGG enrichment results between SZB and PZB. (**Note**: The abscissa represents Rich factor; The ordinate represents the name of KEGG pathways; The color of dots represents P-value; The size of dots indicates the number of differentially accumulated metabolites.)

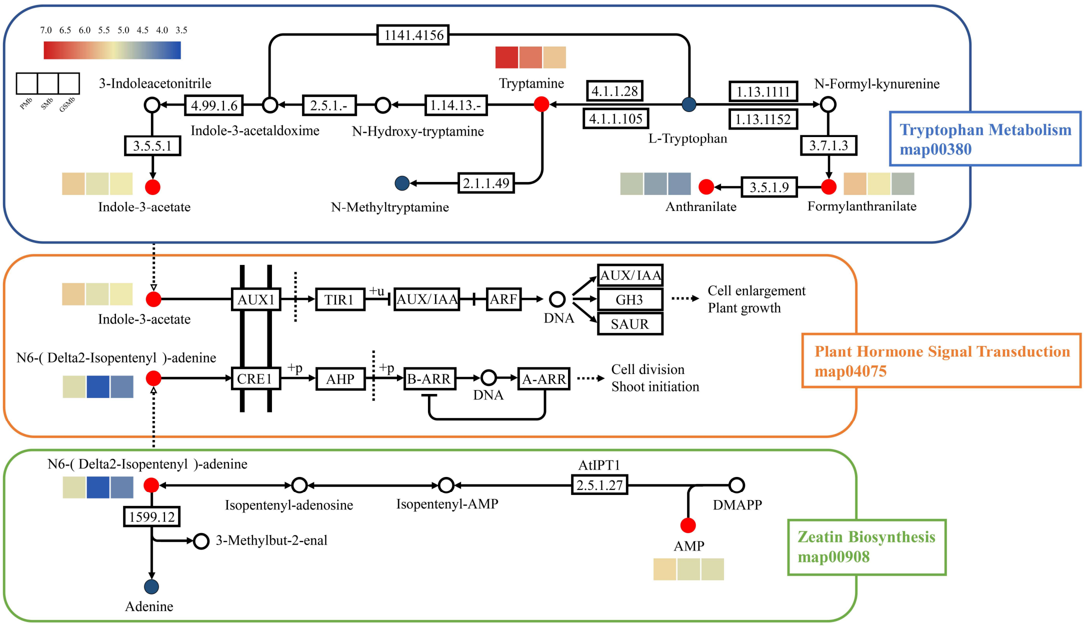

Fig. S13. Differentially Accumulated Metabolites (DAMs) in the Biosynthetic Pathway of Young Stem Bark of *Zanthoxylum bungeanum*. (**KEGG map**: The red dots represent up-regulation, the green dots represent down-regulation, and the blue dots represent no difference. **Heatmap**: The color scale from blue (low) to red (high) represents the foldchange values).

indole-3-acetate, tryptamine, anthranilate, fromylanthranilate,N6-(delta2-isopentenyl)-adenine

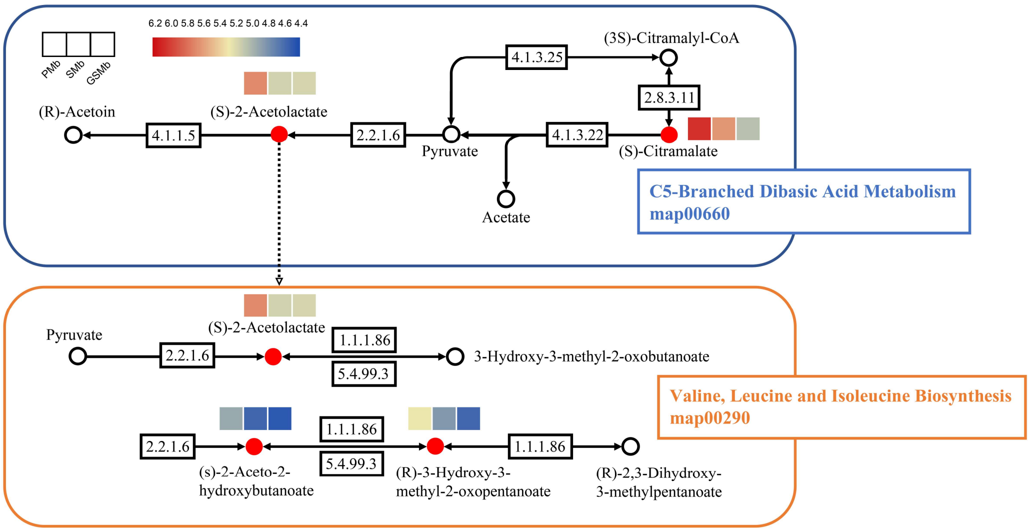

Fig. S14. Differentially Accumulated Metabolites (DAMs) in the Biosynthetic Pathway of Young Stem Bark of Zanthoxylum bungeanum. (KEGG map: The red dots represent up-regulation, the green dots represent down-regulation, and the blue dots represent no difference. Heatmap: The color scale from blue (low) to red (high) represents the foldchange values).

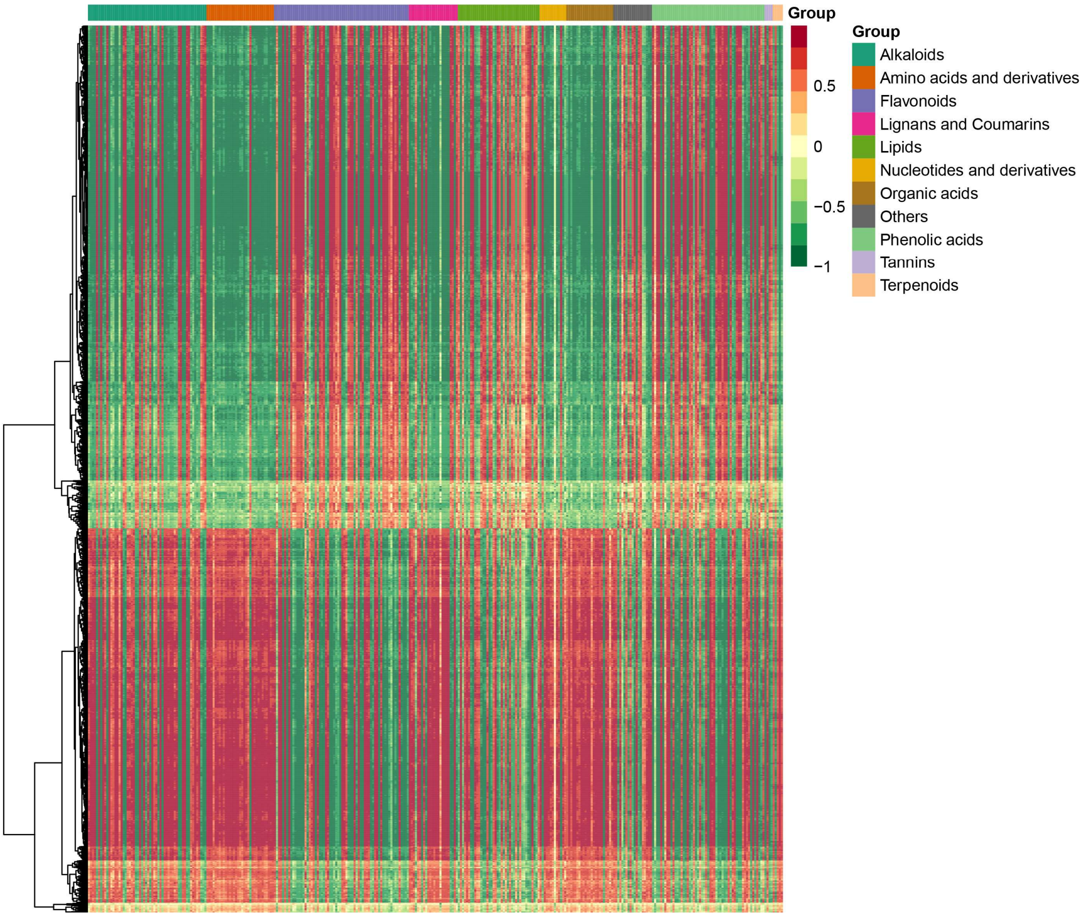

Fig. S15. The correlation heatmap of differentially expressed genes (DEGs) and differentially accumulated metabolites (DAMs) between GSZB and SZB libraries. **Note**: The DEGs and DAMs with PCC (Pearson correlation coefficient) ≥ 0.8 were selected to draw heatmap.

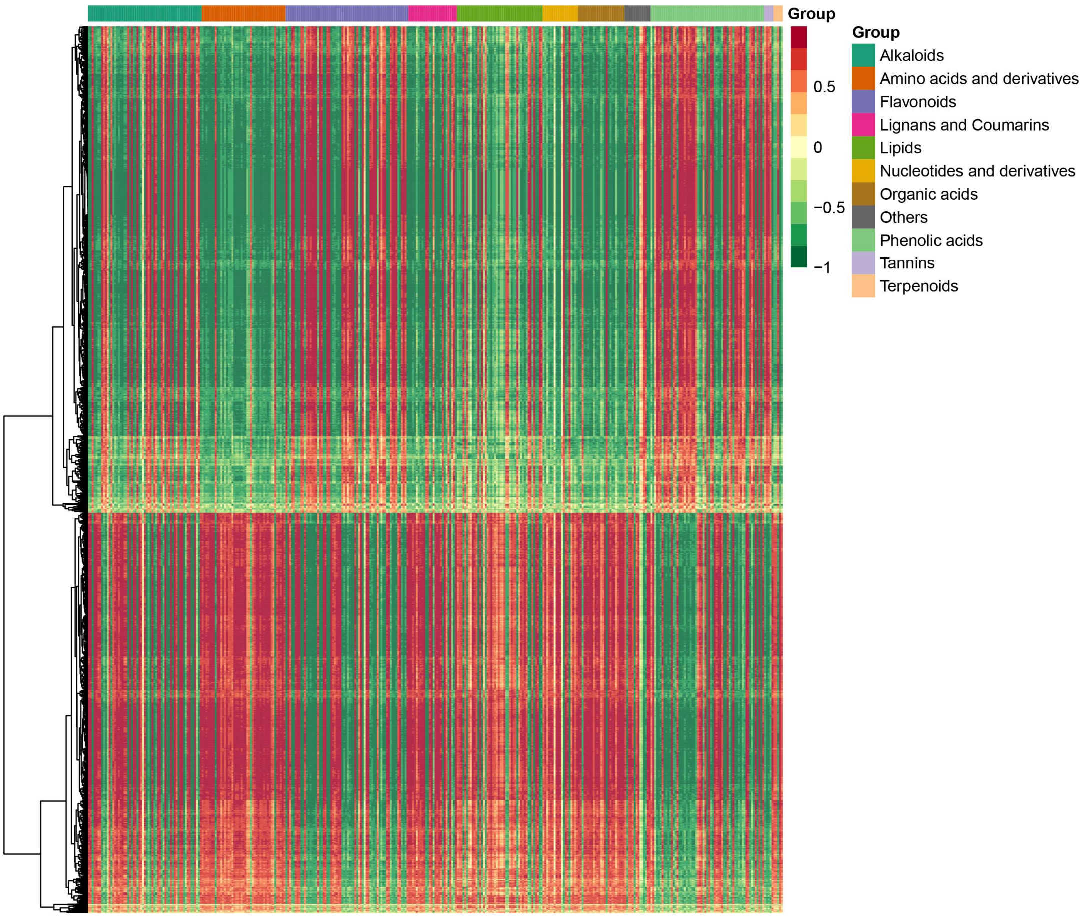

Fig. S16. The correlation heatmap of differentially expressed genes (DEGs) and differentially accumulated metabolites (DAMs) between GSZB and PZB libraries. **Note**: The DEGs and DAMs with PCC (Pearson correlation coefficient) ≥ 0.8 were selected to draw heatmap.
